# Comparison of age-related decline in C57BL/6J and CB6F1J male mice

**DOI:** 10.1371/journal.pone.0306201

**Published:** 2024-12-31

**Authors:** Gerald Yu Liao, Christina Pettan-Brewer, Warren Ladiges

**Affiliations:** Department of Comparative Medicine, University of Washington School of Medicine, Seattle, WA, United States of America; Gyeongsang National University, REPUBLIC OF KOREA

## Abstract

Variability in physical resilience to aging prompts a comprehensive examination of underlying mechanisms across organs and individuals. We conducted a detailed exploration of behavioral and physiological differences between male C57BL/6J and male CB6F1J mice across various age groups (4, 12, 20, 24 months). In behavioral assays, C57BL/6J mice displayed superior performance in rotarod tasks but higher anxiety while CB6F1J mice exhibited a decline in short-term memory with age. Grip strength, long-term memory, and voluntary wheel running declined similarly with age in both strains. Examining physiological phenotypes, C57BL/6J mice exhibited lower body fat percentages across ages compared to CB6F1J mice, though cataract severity worsened with age in both strains. Analysis of cardiac functions revealed differences between strains, with worsening left ventricular hypertrophy and structural heart abnormalities with age in CB6F1J mice along with higher blood pressure than C57BL/6J. Lesion scores showed an age-related increase in heart, kidney, and liver lesions in both strains, while lung lesions worsened with age only in CB6F1J mice. This study underscores the validity of behavioral assays and geropathology assessment in reflecting age-related decline and emphasizes the importance of considering strain specificity when using mouse models to study human aging.

## Introduction

Aging is a universal phenomenon characterized by a gradual decline in physiological function and an increased susceptibility to disease, posing significant challenges to human health. A complete understanding of the underlying mechanisms of aging is therefore paramount for the development of effective treatments against age-related diseases, which are major contributors to morbidity and mortality in humans [1]. Given that between-individual and between-organ heterogeneity in health suggests variability in response to physical stress [2], the documentation of resilience across the lifespan, starting at a relatively young age in response to physical stress, offers valuable insights into individual health trajectories and the promotion of healthy aging.

However, the complexities inherent in human-based research, including ethical, social, and cost considerations, coupled with the entered human lifespan, necessitate alternative approaches to studying aging. Animal models have emerged as pragmatic tools for investigating age-related disease progression and treatment responses. While no single animal model can fully replicate all aspects of human aging, a thorough understanding of the characteristics of specific models and judicious interpretation of results can facilitate targeted investigations into critical facets of age-related diseases and their treatments [3].

Mice, due to their relatively short lifespan and modifiable genetic makeup, are among the most widely used animal models in aging research. Characterized by its widespread availability and well-documented genetic, behavioral, and cognitive background, the inbred C57BL/6J strain stands out as a prominent model in the study of aging and age-related diseases [1]. In addition, a cross between C57BL/6J and Balb/cJ, designated as the CB6F1J mice and characterized by its more heterogeneous genetic background compared to the parenteral inbred strains, has also been validated [4]. Both are readily available from the National Institute on Aging Aged Rodent Colony. Nevertheless, few studies have assessed differences in age-related changes in behavior and cognition between the two strains [5, 6], and no studies have conducted a comprehensive examination of differences based on age-related behavioral, physiological, and geropathological differences.

In the present study, we address this gap by conducting a series of behavioral assays (i.e., rotarod performance, radial water tread maze, grip strength, open-field test, voluntary wheel running), physiological phenotypes (i.e., cataract severity, body composition assessment, cardiac functions (left ventricular hypertrophy, cardiac reserve, ejection fraction, structural heart abnormalities), blood pressure), and geropathological assessments (i.e., heart, kidney, lung, liver) in C57BL/6J and CB6F1J male mice at different ages. By systematically analyzing various parameters, we aimed to elucidate the differences in aging characteristics between these strains and provide comparative insights as models of aging and age-related diseases.

## Materials and methods

### Animals

CB6F1J (C57BL/6J X Balb/cJ F1 cross) (20 at 4 months-, 20 at 12 months-, 20 at 20 months-, and 29 at 28 months-old) and C57BL/6J male mice (20 at 4 months-, 20 at 12 months-, 20 at 20 months-, and 27 at 28 months-old) were obtained from the National Institute on Aging Aged Rodent Colony, contracted by Charles River, Inc. Mice were housed in a specific pathogen free mouse facility at the University of Washington (UW) main campus in Seattle, WA. The status of the room was monitored under the guidance of the Rodent Health Monitoring Program within the purview of the UW Department of Comparative Medicine. Mice were group housed, up to five per cage, given nestlets (Ancare Corp, Bellmore, NY) for environmental enrichment and fed *ad lib* standard rodent chow obtained from Picolab (Rodent Diet 20, 5053, Irradiated, containing corn, soybean, wheat, fish meal, and a vitamin-mineral mixture).

Mice were acclimated for two weeks before starting test procedures. All procedures were approved by the University of Washington IACUC (Animal Care and Use Committee).

### Behavioral assays

#### Rotarod performance

Agility, a measure of balance and coordination, was assessed using a Rotamax 4/8 rotating bar machine (Columbus Instruments, Inc) [5, 7]. The machine tested the ability of mice to maintain walking speed on a rotating bar, with individual lanes separated to prevent visual or physical interactions. Seven photobeams were embedded in each lane of the enclosure with a software recording the photobeam breaks during the task. Once the animal falls from the rotarod, the final time is recorded because of the absence in beam breaks. The initial speed was set to 0 RPM and gradually increased by 0.1 RPM/s over a 5-minute duration until 40 rpm, when all mice fell off and were detected by a sensor. The time in seconds was recorded for each mouse over three trials and the mean time of the three trials per mouse was recorded.

#### Memory assessment

A radial water tread maze was used to assess short-term and long-term memory [8]. The maze consisted of a circular basin with nine holes, eight decoys leading to dead ends, and one escape hole leading to a dark safety box equipped with a heating pad to simulate a standard mouse cage. The basin contained approximately one inch of water and an overhead light placed above the cage as an escape incentive. Mice spent a maximum of 3 minutes per trial in the maze, with those that were stationary for greater than 10 seconds brought back to the center of the apparatus. Mice had three trials in the maze for four consecutive days of training, followed by testing on the fifth day and retesting on the twelfth day.

#### Grip strength assessment

Forelimb strength, a measure of frailty in elderly humans was measured using a Grip Strength Meter [9, 10]. Each mouse was positioned horizontally with forepaws on a metal grip bar (Columbus Instruments, Inc), and the mouse was pulled back at a uniform rate until releasing the bar. The machine recorded the maximum force exerted by the mouse for a total of five trials. Mice were weighed on the test day and peak force was expressed relative to body weight to normalize grip strength measurements.

#### Voluntary wheel running assessment

Total distance ran over three days was measured with a running wheel added to a standard mouse cage [11]. Mice were individually housed in standard cages with a slanted running wheel wirelessly connected to a computer (Med Associates, Inc). There was a two-day acclimation period with the wheels locked and on the third day the wheel was unlocked, and data collection began. Running distances were continuously monitored over a 72-hour period with total distances ran every minute recorded in kilometers.

#### Anxiety assessment

An open-field photobeam testing system (OFT) (Columbus Instruments, Inc) was employed to evaluate anxiety-related behaviors of mice in a novel environment as previously described [12]. The apparatus simulates a standard mouse cage, featuring a clear rectangular container and infrared beams arranged in a grid pattern, three horizontally and four vertically. The open-field photobeam system was configured with two sets of infrared beams to measure both lateral and vertical activity. Beam breaks, which occurred when mice crossed an infrared beam, were counted for each activity. The data collected were subsequently categorized into two distinct zones: the central and peripheral areas of the container. This categorization allowed for the assessment of anxiety levels based on preference for exploring specific regions. Increased time spent in the central area suggested reduced anxiety, whereas a preference for peripheral regions suggested heightened anxiety. Each mouse was placed inside the testing container for a period of five minutes. This standard duration ensured consistent evaluation of anxiety-related behaviors and minimized potential habituation effects or stress-related responses.

### Physiological phenotypes

#### Cataract assessment

Recognized as a marker of visual function in previous studies [13–15], cataract formation was assessed using slit-lamp ophthalmoscopy. Both eyes of each mouse were examined and averaged to ensure comprehensive assessment of cataract progression. Based on lens opacity, the degree of cataract progression was graded on a scale from 0 to 4, with increments of 0.5. A score of 0 represented complete clarity of the lens, while a score of 4 indicated the presence of a mature cataract occupying the entire lens.

#### Body composition assessment

Changes in body composition, even in the absence of body weight changes, is a well-documented factor of aging [16]. Body composition, including total body fat and lean mass, were assessed using noninvasive quantitative magnetic resonance (QMR) imaging (Echo Medical System).

#### Cardiac function assessment

Echocardiography, a non-invasive procedure, was employed to assess systolic and diastolic function in mice [17]. The Siemans Acuson CV-70 system was utilized, employing standard imaging planes including M-mode, conventional, and Tissue Doppler imaging. Parameters such as left ventricular mass index (LVMI), left atrial (LA) dimension, end-diastolic and end-systolic dimensions, LV fractional shortening, Ea/Aa (diastolic function) measured by tissue Doppler imaging of the mitral annulus, and Myocardial Performance Index (MPI) were measured, as previous described [18]. Additionally, blood pressure measurements were obtained and cross-correlated with echocardiographic functional parameters to provide comprehensive cardiac function assessment.

### Geropathology

Mice in all groups were decapitated after euthanasia via 5 min carbon dioxide inhalation approved by Institute/Center (IC) Animal Care and Use Committee (ACUC). Necropsy was performed after euthanasia with a postmortem time of 5 min. Systematic sections of heart, lung, liver, and kidney were flash frozen and stored at -80 degrees Celsius. Sections of the same organs were also fixed in 10% buffered formalin for 48 h then blocked in paraffin wax through University of Washington Department of Comparative Medicine’s Histology Lab and subsequently sectioned. Sections of 4 um fixed tissues were stained with hematoxylin and eosin for geropathology grading by two board certified veterinary pathologists in a blinded manner according to published guidelines [19] using a Nikon Eclipse Ci microscope with a 20x objective. The geropathology scores were tabulated into a composite lesion score (CLS) for each of four major organs: heart, lungs, liver, and kidney, and recorded for each animal. The CLS was calculated by adding the severity score (from 1 to 4) of each lesion in an organ for each mouse in a cohort, and then adding the total lesion score of each organ for all mice in a specific cohort and dividing by the number of mice in that cohort. Therefore, the CLS is a standardized score that can be used statistically to compare lesion severity among different cohorts.

### Statistical analysis

All data was grouped according to strain and age. A Shapiro-Wilk test was used to assess data under each group to determine whether there was a normal distribution. Two-tailed student’s t-test was used to compare between results from each treatment cohort for normally distributed data while the Mann-Whitney U test was used to determine significant differences between two groups when the data was not normally distributed. For geropathology, scoring data was the average lesion score from two pathologists analyzed by cohort using the two-tailed student’s t-test. Mean values with standard error bars (SEM) are presented by group in the figures. Quantitative interval outcomes collected from both pathologists were normalized for central tendency analysis. Correlation analysis between treatment cohorts, strains and lesion scores were done by two-way analysis of variance (ANOVA). All statistical tests were conducted at 0.05 significance level and adjusted for multiple pairwise comparisons using the Bonferroni method. All statistical analyses were performed using GraphPad prism (version 10.0.3).

## Results

### Rotarod performance was age-dependent, with C57BL/6J mice performing better

**Fig 1:**
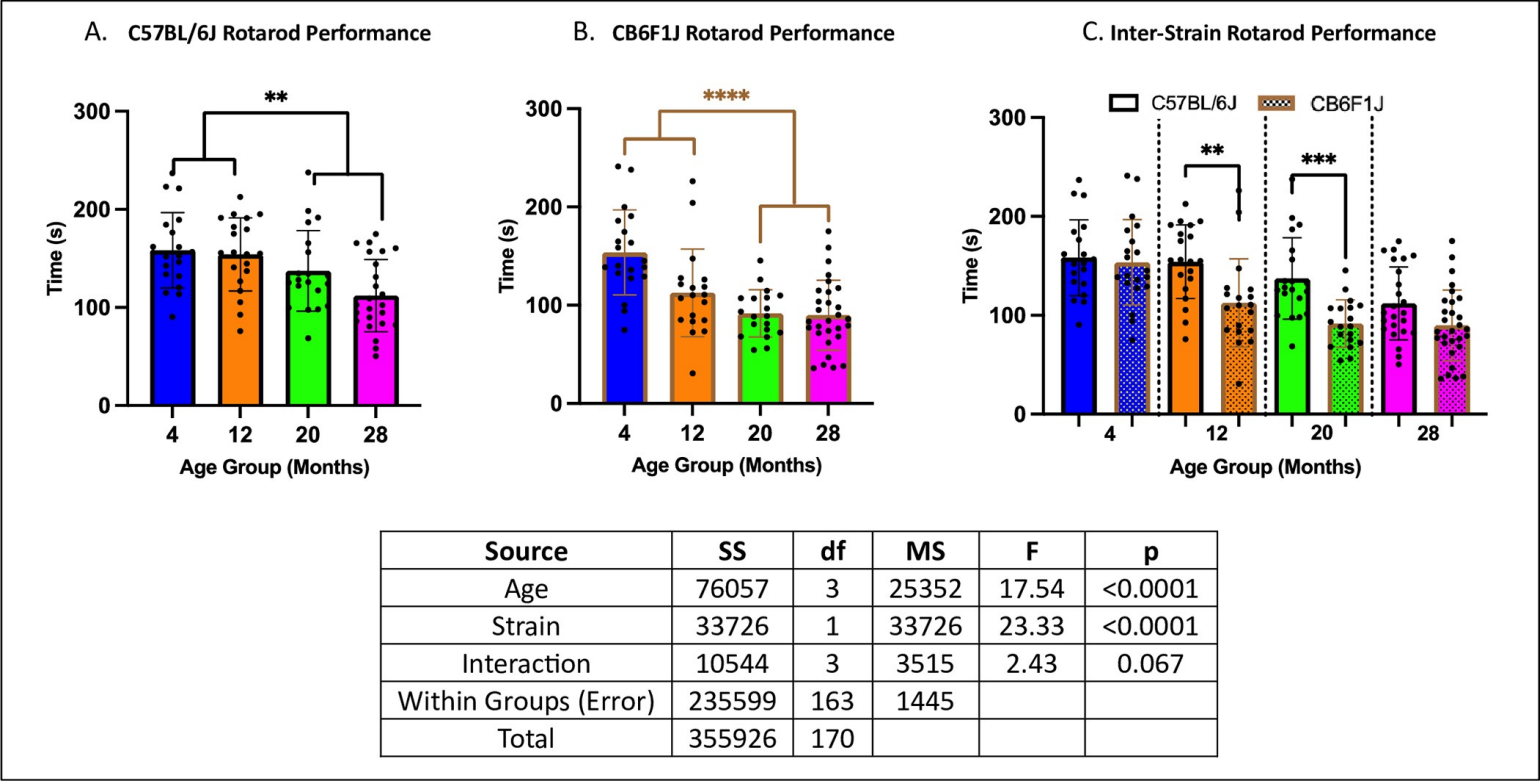
Rotarod performance. **A.** CB6F1J mice at 20 and 28 months of age exhibited significant differences when compared to 4 and 12-month-old cohorts and showed a negative association between performance and age. **B.** C57BL/6J mice at the same age groups also showed statistically significant age-related decrease in performance as seen in CB6F1J mice. **C.** Inter-strain comparisons across age groups demonstrated greater performance in C57BL/6J compared to CB6F1J mice (** p < 0.01, ***p < 0.001, ****p < 0.0001, N = 19-29/cohort).

Older male mice (20 to 28 months) performed worse compared to younger mice (4 to 12 months) in both strains (p’s < 0.01) (Fig 1A and 1B). Additionally, C57BL/6J mice consistently outperformed CB6F1J mice (Fig 1C) (p’s < 0.01).

### Memory in the radial water tread maze was age- and strain-dependent

**Fig 2:**
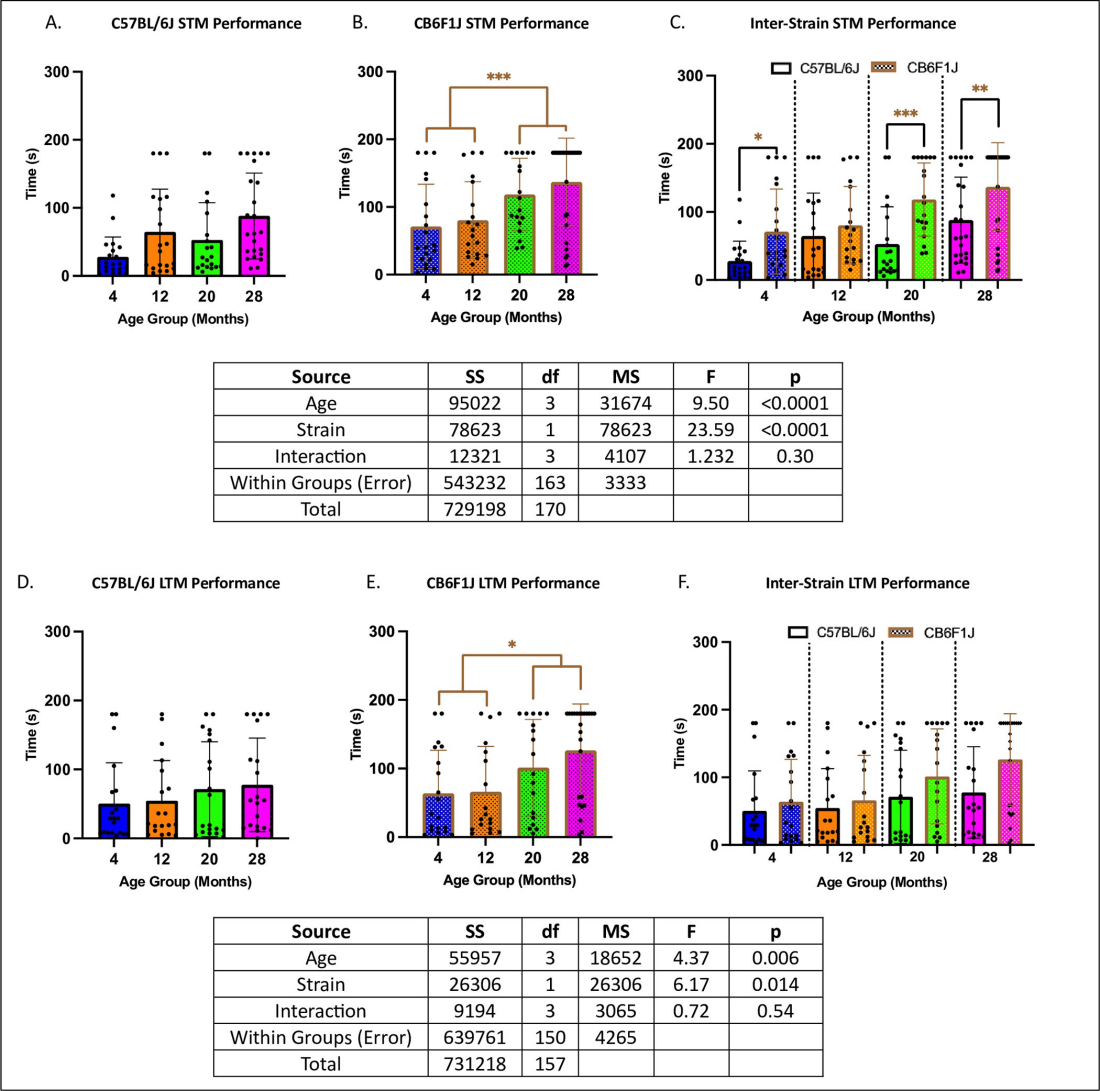
Memory performance. **A/B. Short-term memory (STM):** C57BL/6J mice at 20 and 28 months of age did not exhibit significant differences when compared to their younger counterparts, while CB6F1J mice at 20 and 28 months of age exhibited significant differences when compared to their younger counterparts. **C. STM:** C57BL/6J mice performed better than CB6F1J across age groups. **D/E. Long-term memory (LTM):** Similar to STM, C57BL/6J mice did not exhibit age-related differences while CB6F1J did. **F. LTM:** C57BL/6J mice performed similarly to CB6F1J across age groups (*p < 0.05, **p < 0.01, ***p < 0.001, N = 19-29/cohort).

In C57BL/6J mice, memory performance did not differ significantly between older mice (20 to 28 months) and younger mice (4 to 12 months) (p’s > 0.05) (STM: Fig 2A, LTM: Fig 2D). However, in CB6F1J mice, older cohorts exhibited impaired memory compared to younger cohorts (p’s < 0.05) (STM: Fig 2B, LTM: Fig 2E). Additionally, C57BL/6J mice generally demonstrated better STM compared to CB6F1J mice (p’s < 0.05) (Fig 2C), while no significant differences were observed in LTM between the strains (p’s > 0.05) (Fig 2F).

### Grip strength & wheel running performance decreased with age in both CB6F1J & C57BL/6J mice

**Fig 3:**
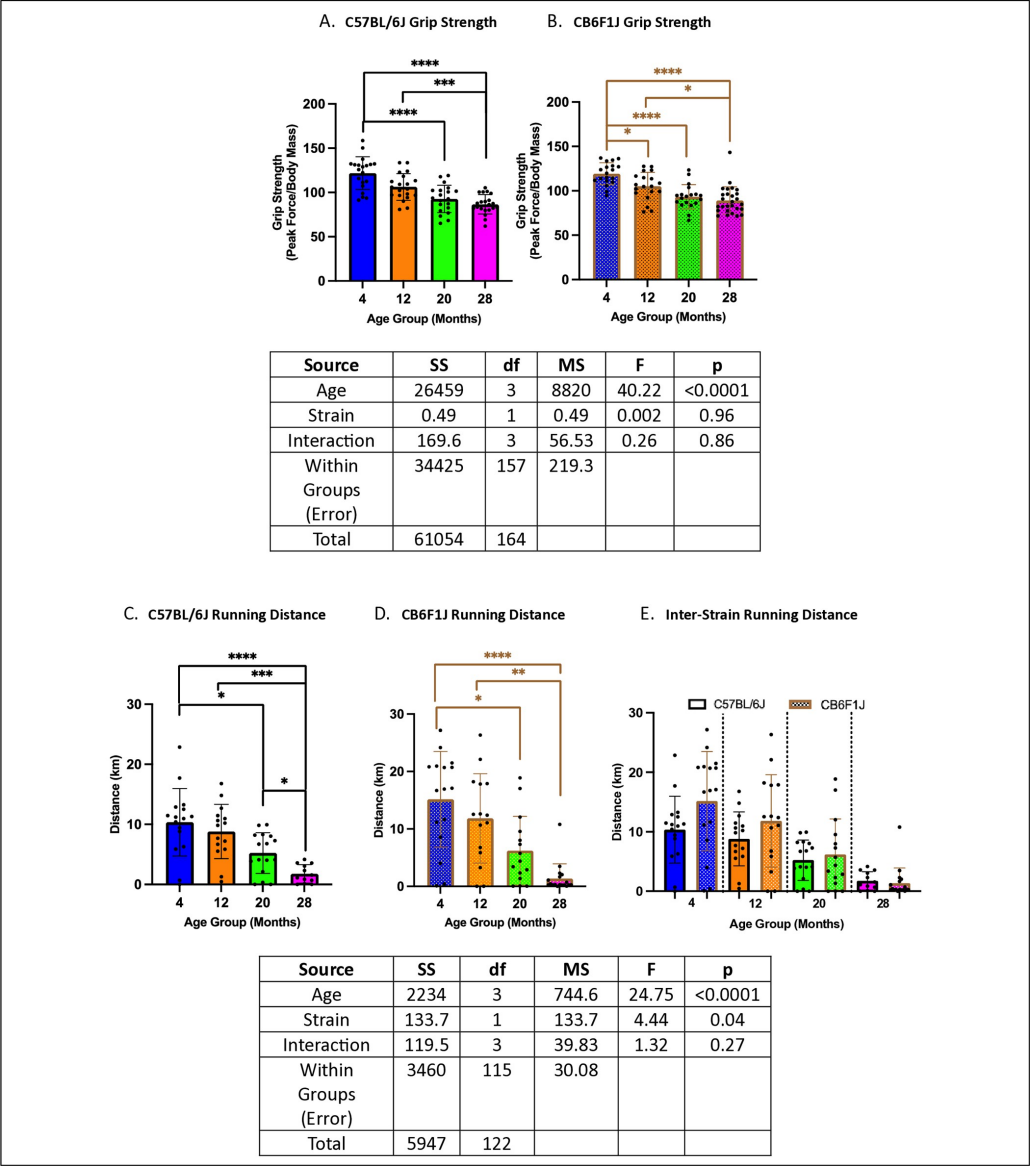
Grip strength & running distance. **A/B.** Both C57BL/6J and CB6F1J mice exhibited decreased grip strength with age. **C/D.** Both C57BL/6J and CB6F1J mice exhibited decreased running distance with age. **E.** No significant differences in running distance between strains were detected among all age groups (*p < 0.05, **p < 0.01, ***p < 0.001, ****p < 0.0001, N = 19-27/cohort for grip strength, N = 12-18/cohort for running distance).

Across all age groups, both strains exhibited similar performance, with no significant differences detected between them (p’s > 0.05). (Fig 3E) Specifically, grip strength was significantly lower in older cohorts compared to younger ones (p’s < 0.05) (Fig 3A and 3B). Similarly, running distance showed a decline in older mice across both strains (p’s < 0.05) (Fig 3C and 3D).

### Older C57BL/6J mice had higher anxiety compared to CB6F1J Mice

**Fig 4:**
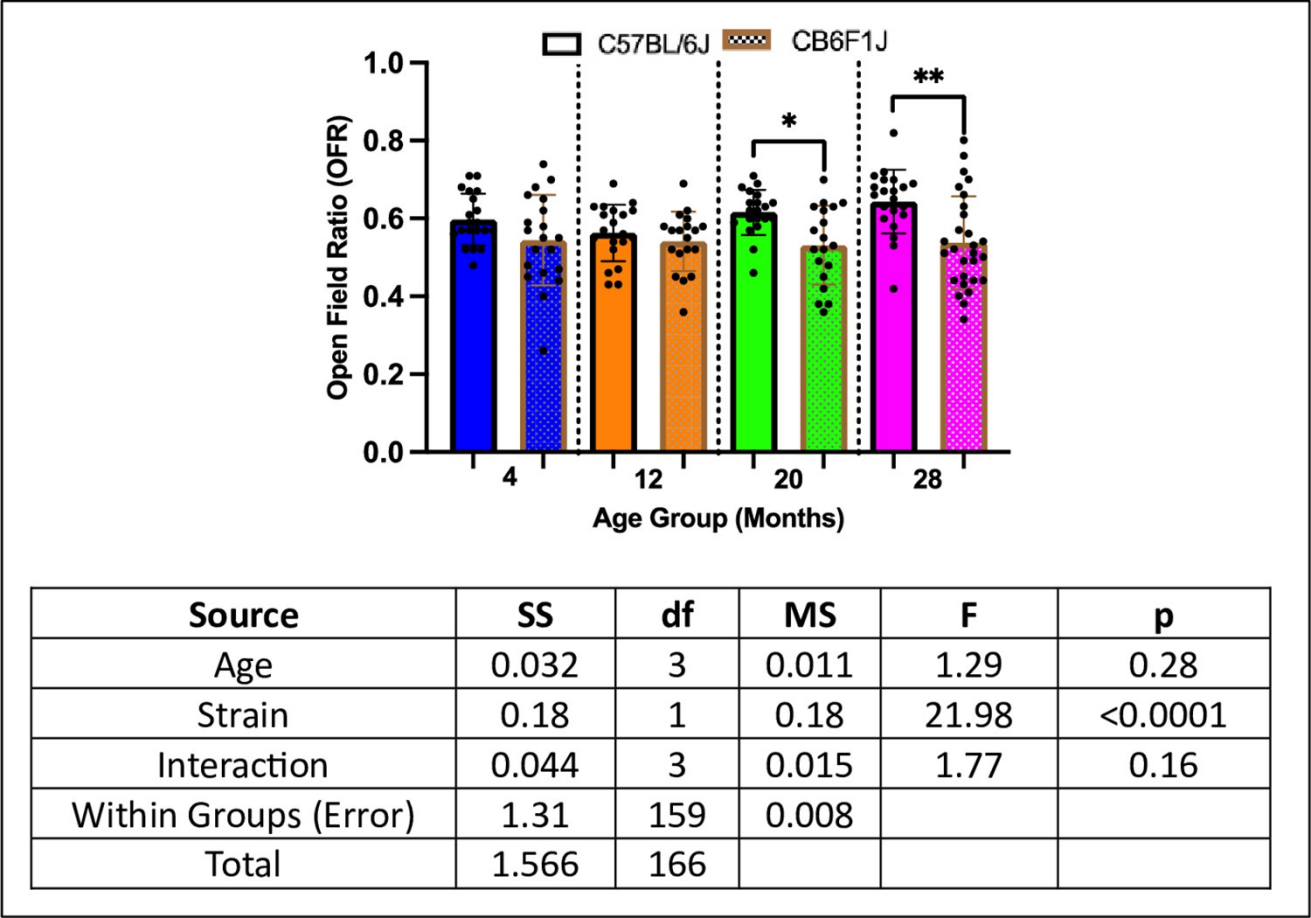
Anxiety scores. C57BL/6J and CB6F1J mice had similar anxiety scores at the 4- and 12- month cohorts although C57BL/6J mice had significantly higher anxiety at 20- and 28-months of age compared to CB6F1J mice (*p < 0.05, **p < 0.01, N = 19-28/cohort).

Older C57BL/6J mice at the 20- and 28- month cohorts had higher anxiety compared to their CB6F1J counterparts (p’s < 0.05) while no differences were observed between the younger cohorts at 4- and 12- months (p’s > 0.05) (Fig 4).

### Cataract severity increased in an age-dependent manner in both C57BL/6J and CB6F1J mice

**Fig 5:**
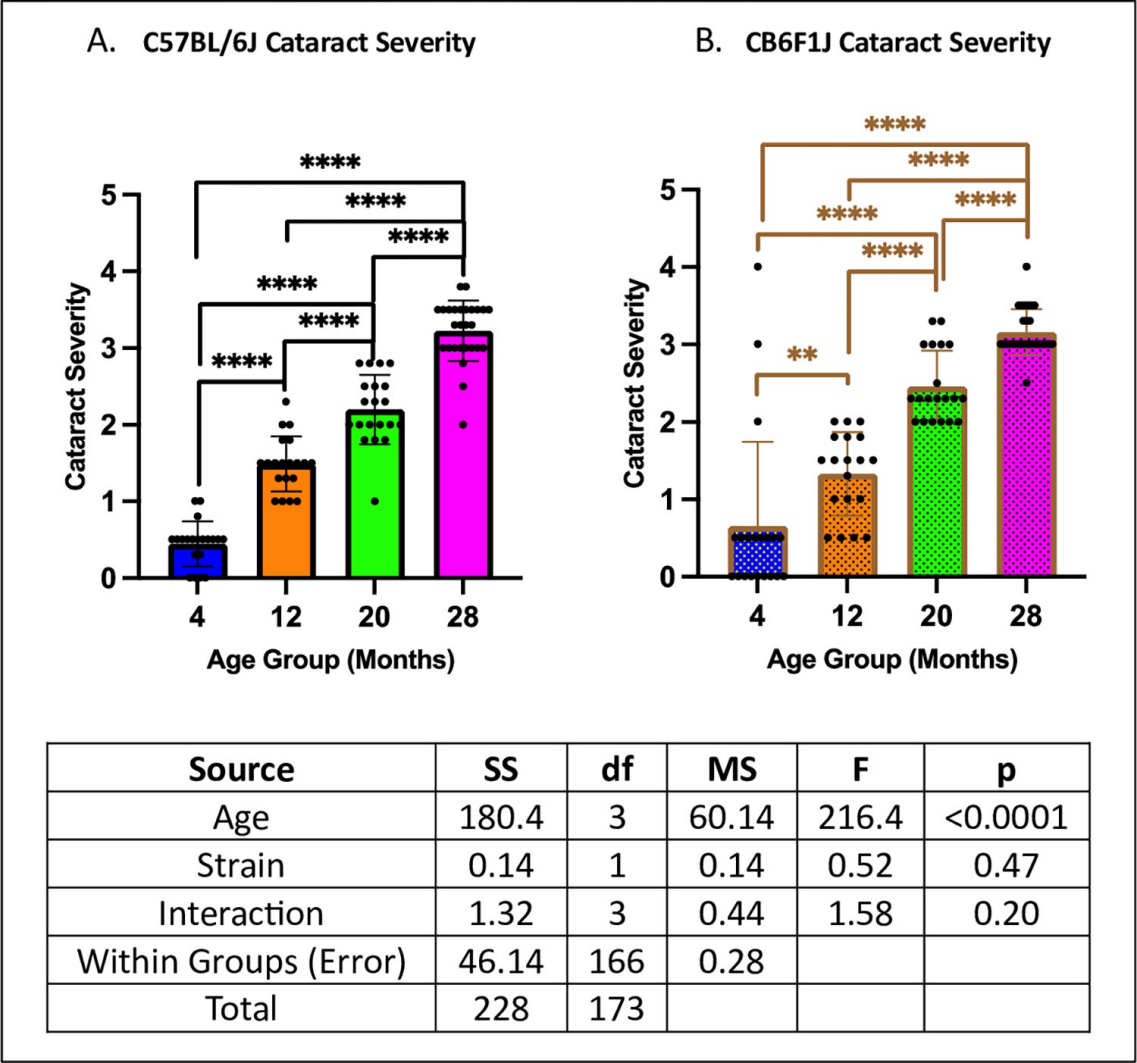
Cataract severity. **A/B.** Both C57BL/6J and CB6F1J mice exhibited greater cataract severity with age (**p < 0.01, ****p < 0.0001, N = 19-28/cohort).

Both strains exhibited similar performance across age groups (p’s > 0.05), with cataract severity progressively increasing with age (p’s < 0.01) (Fig 5A and 5B).

### Body composition was age- and strain-dependent

**Fig 6:**
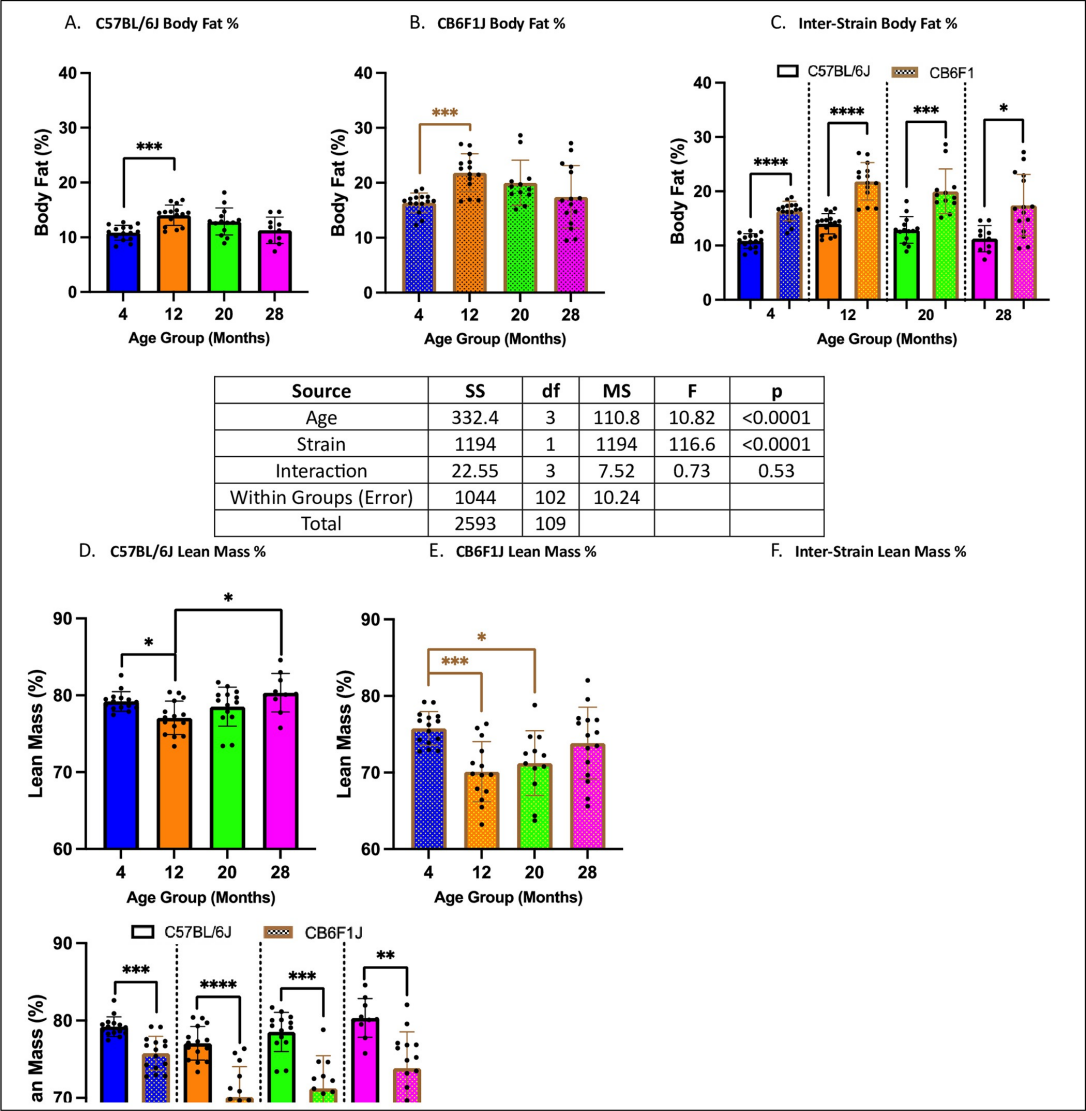
Body composition assessment. **A/B.** C57BL/6J and CB6F1J mice had relatively stable body fat with only a significant difference between 4- and 12- month cohorts. **C.** Overall, CB6F1J mice had significantly higher body fat than their C57BL/6J counterparts across age groups. **D/E.** C57BL/6J and CB6F1J mice showed age-related differences in lean body mass. **F.** Overall, C57BL/6J mice had significantly higher lean body mass compared to their CB6F1J counterparts across age groups (*p < 0.05, **p < 0.01, ***p < 0.001, ****p < 0.0001, N = 10-15/cohort).

Significant differences in body fat were observed only between the 4- and 12-month cohorts in both strains (p’s < 0.001) (Fig 6A and 6B). However, CB6F1J mice consistently exhibited higher body fat levels compared to C57BL/6J mice across all age groups (p’s < 0.05) (Fig 6C). Additionally, differences in percent lean body mass were evident in both strains across age groups (p’s < 0.05) (Fig 6D and 6E), with C57BL/6J mice consistently displaying higher percent lean body mass compared to CB6F1J mice across all age groups (p’s < 0.01) (Fig 6F).

### Cardiac functions were age- and strain-dependent

**Fig 7:**
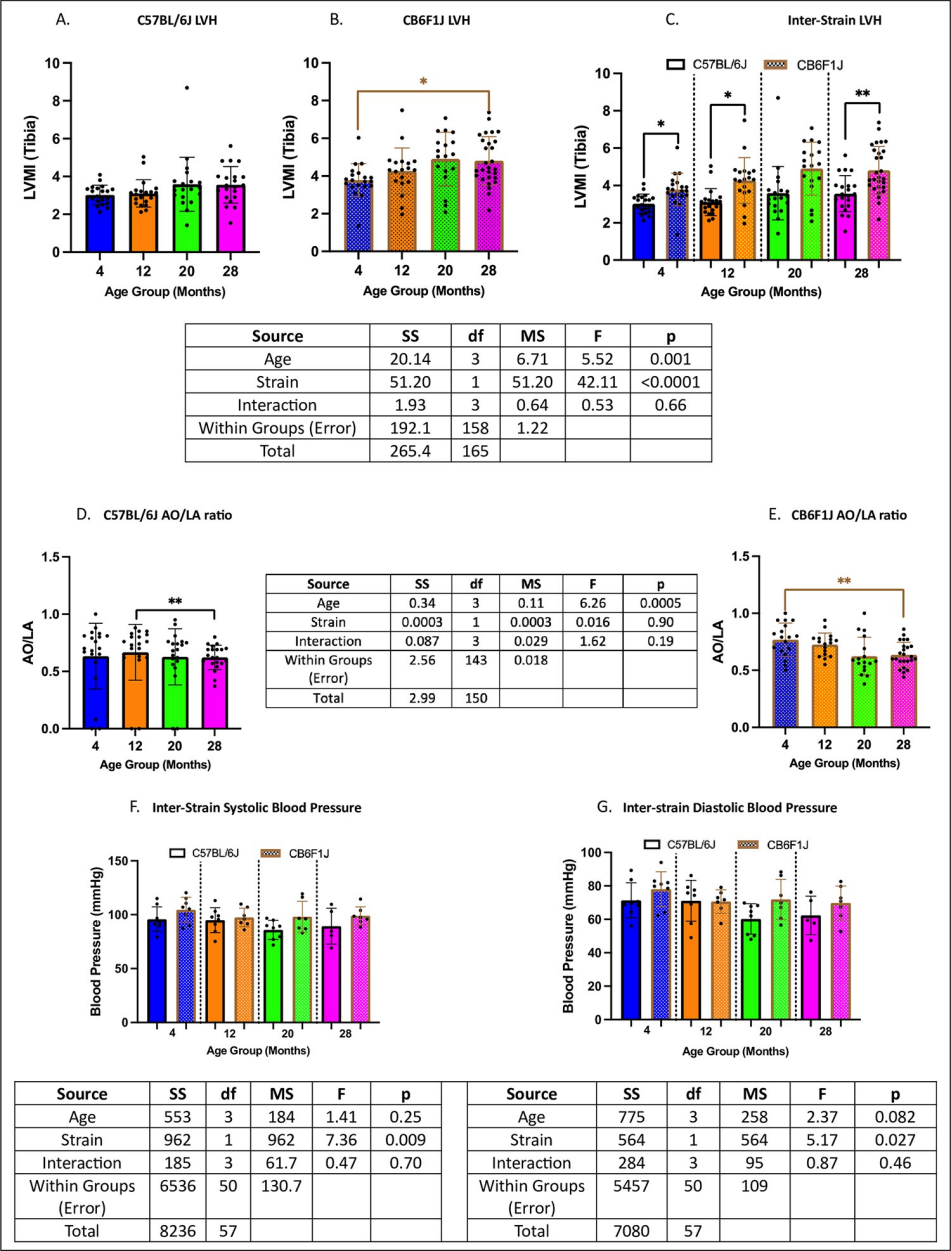
Cardiac function. **A.** C57BL/6J mice did not show age-related changes in LVMI (tibia). **B.** 28-month-old CB6F1J mice had significantly lower LVMI (tibia) compared to their 4-month-old counterparts. **C.** CB6F1J mice had generally higher LVMI (tibia) than C57BL/6J mice. **D/E.** Age-related changes in AO/LA ratio were observed in both C57BL/6J and CB6F1J mice, with lower ratios in the older age groups. **F/G.** Neither strain experienced age-related decreases in systolic/diastolic blood pressure, while CB6F1J mice trended higher readings compared to their C57BL/6J counterparts (*p < 0.05, **p < 0.01, N = 18-21/cohort for LVMI (tibia), N = 17-24/cohort for AO/LA ratio, N = 5-8/cohort for blood pressure).

Left ventricular mass index normalized to tibial length (LVMI (tibia)) was used as a measure of left ventricular hypertrophy (LVH). No age-related changes were observed in C57BL/6J mice (p’s > 0.05) (Fig 7A), with differences detected only between the 4- and 28-month CB6F1J cohorts (p < 0.05) (Fig 7B). Inter-strain comparisons revealed significantly higher LVMI (tibia) in CB6F1J mice compared to their C57BL/6J counterparts (p’s < 0.05) (Fig 7C).

The aortic root to left atrial ratio (AO/LA) served as another measurement of structural heart abnormalities alongside LVMI (tibia). Differences between older and younger cohorts were observed in both C57BL/6J and CB6F1J strains, with lower AO/LA ratio detected in the older cohorts (p’s < 0.01) (Fig 7D and 7E).

Additionally, average diastolic and systolic blood pressures were measured as variables of cardiac function. There was a trend towards higher systolic blood pressure in the CB6F1J mice compared to the C57BL/6J mice across all age groups (p < 0.01) (Fig 7F). Similarly, diastolic blood pressure showed a slight but consistent increase in CB6F1J mice relative to C57BL/6J mice (p < 0.05) (Fig 7G). No associations with age or strain were found for measures of left ventricular diastolic dysfunction (i.e., Ea/Aa ratio, IVCT T1, IVRT T3), cardiac reserve (i.e., MPI), or changes in ejection fraction (i.e., FS %).

### Geropathology of the heart, liver, and kidney were age-dependent in both CB6F1J and C57BL/6J mice while CB6F1J had more severe lung lesions

**Fig 8:**
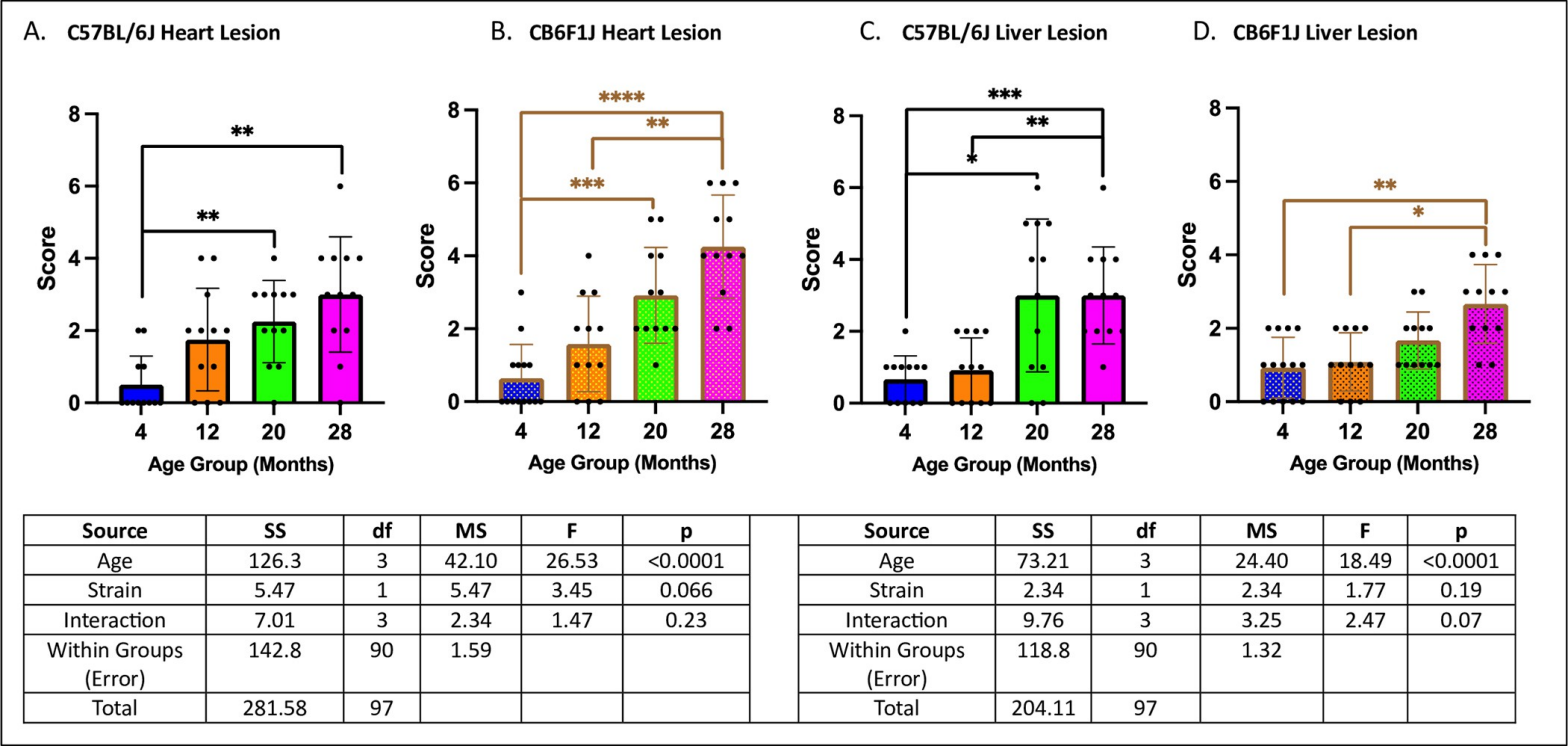
Heart/liver lesion scores. **A/B.** Both C57BL/6J and CB6F1J exhibited age-related worsening heart lesion severity. **C/D.** Both C57BL/6J and CB6F1J mice exhibited age related worsening liver lesion severity (*p < 0.05, **p < 0.01, ***p < 0.001, ****p < 0.0001, N = 12-14/cohort).

There were age-related increases in heart lesion severity in both CB6F1J and C57BL/6J strains (p’s < 0.01) (Fig 8A and 8B). Similarly, age-related increases in liver lesion severity were observed in both strains (p’s < 0.05) (Fig 8C and 8D).

**Fig 9:**
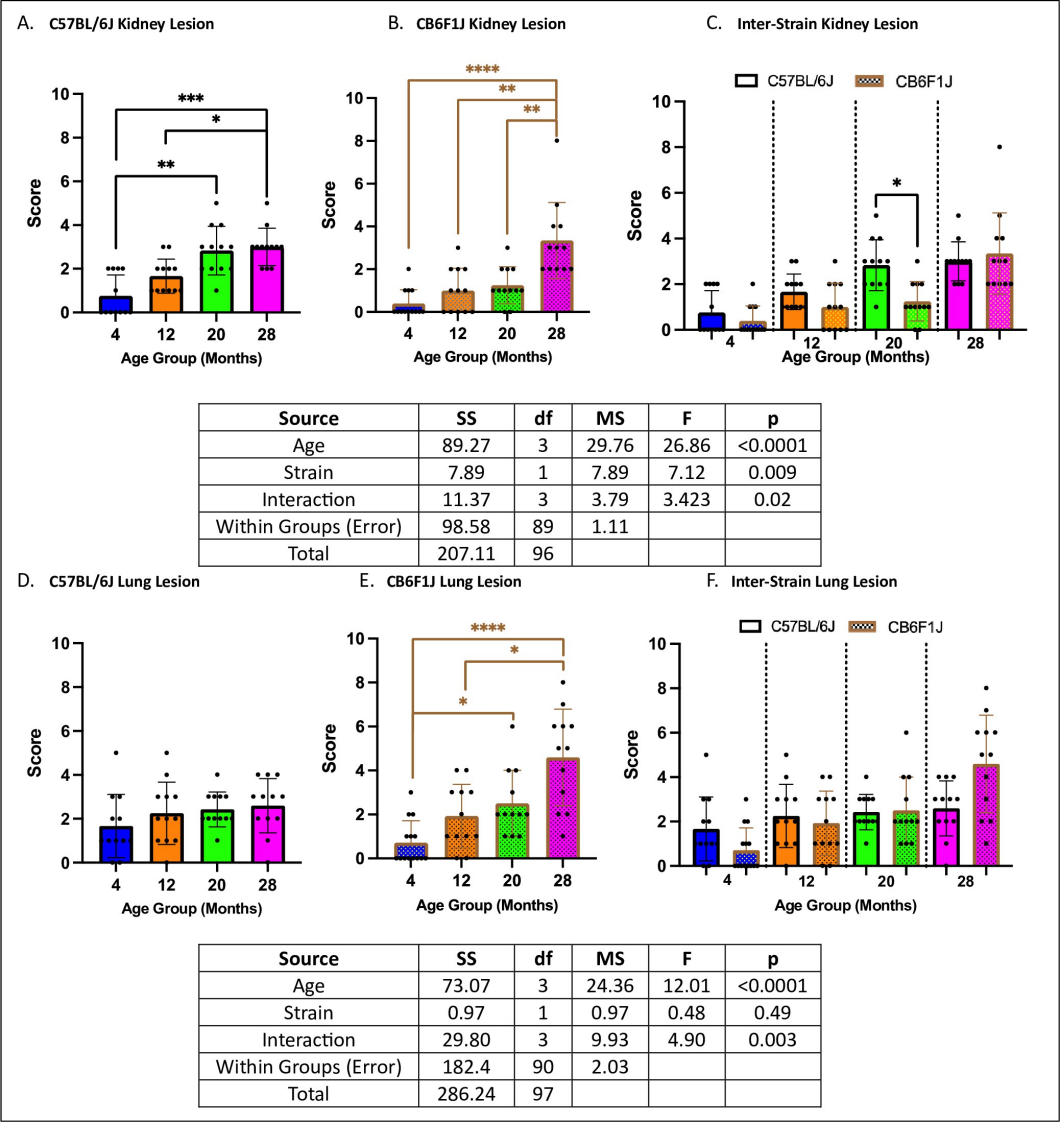
Kidney/lung lesion scores. **A/B.** Both C57BL/6J and CB6F1J mice exhibited age-related increases in kidney lesion severity. **C.** Only 20-month-old C57BL/6J mice had significantly higher degrees of kidney lesion compared to their age-adjusted CB6F1J counterparts. **D.** C57BL/6J mice did not have significant age-related differences in lung lesion severity. **E.** CB6F1J mice had age-related increases in lung lesion severity. **F.** C57BL/6J and CB6F1J mice had similar amounts of lung lesion severity across all age groups (*p < 0.05, **p < 0.01, ***p < 0.001, ****p < 0.0001, ns = not significant (p > 0.05), N = 12-14/cohort).

Both C57BL/6J and CB6F1J mice exhibited increased kidney lesion severity with age (p’s < 0.05) (Fig 9A and 9B), with 20-month-old C57BL/6J mice showing more severe kidney lesions than their CB6F1J counterparts (p < 0.05) (Fig 9C). Additionally, there was an age-related increase in lung lesion severity in CB6F1J mice (p’s < 0.05) but not in C57BL/6J mice (p’s > 0.05) (Fig 9D and 9E), without any differences between strains (p’s > 0.05) (Fig 9F).

**Fig 10:**
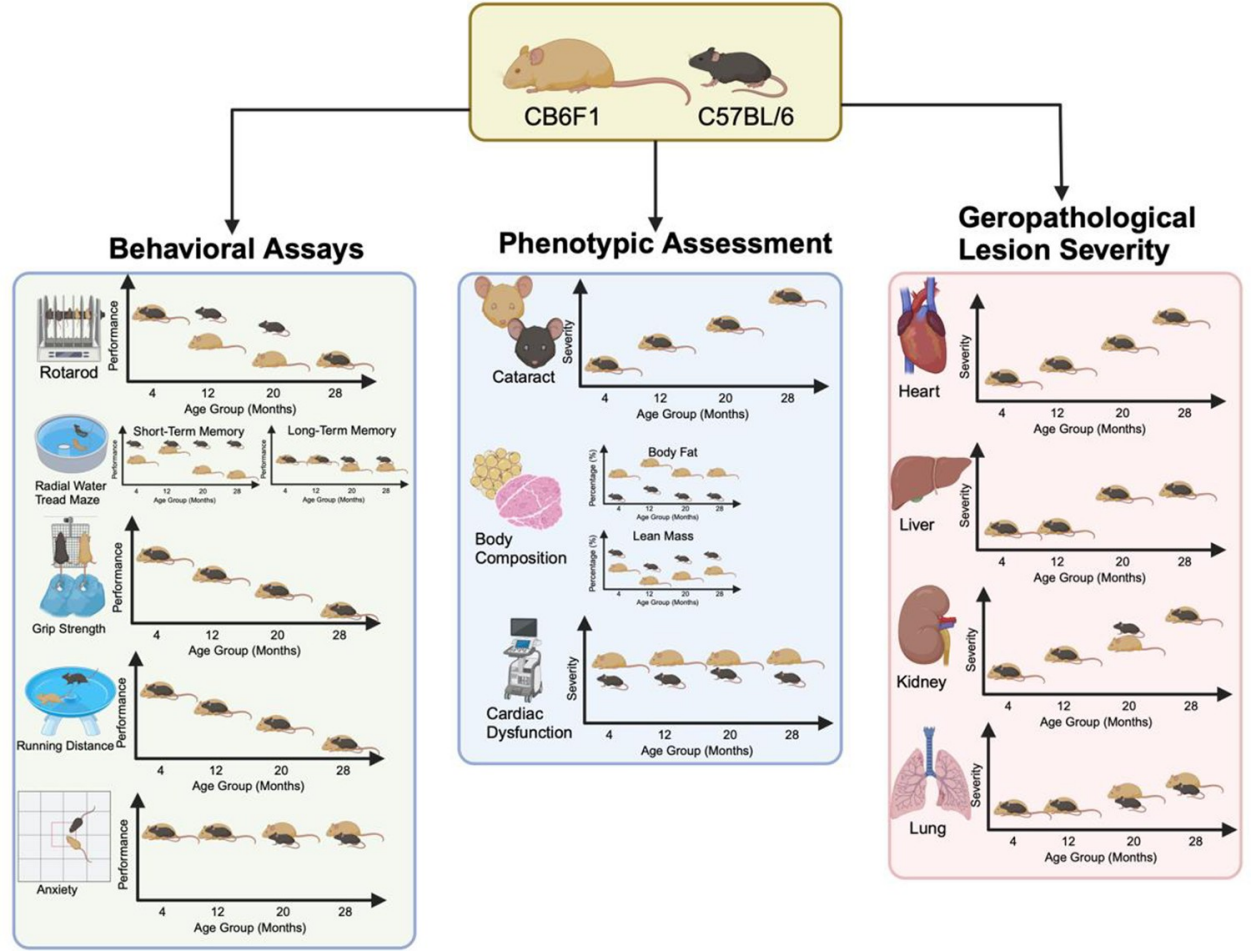
Summary of results. Featuring a visual comparison between the two mouse strains, each strain is depicted alongside corresponding images illustrating the specific comparisons made. Differences between te strains are represented by the vertical distance on the axes.

Overall comparison of age- and strain-related differences from the results are summarized in Fig 10.

## Discussion

Analysis of inter-strain differences and similarities of behavioral, physiological, and geropathological assays between CB6F1J and C57BL/6J mice revealed insights into the relationships between aging and strain-specificity.

As a characteristic of aging across species is a decline in muscle function, which is evidenced by reduced strength and muscle mass with age [20–22], the ability to maintain neuro-muscular performance is therefore pivotal for extending health-span during aging. To obtain a comprehensive profile of muscular ability while minimizing confounding variables such as motivation and sensory deficits, we assessed rotarod performance, grip strength, and wheel running tasks. Both 12- and 20-month-old C57BL/6J mice outperformed CB6F1J counterparts on the rotarod task despite age-related performance decline across tasks. While cataract formation, a well-documented consequence of aging [23] shown to increase in severity with age in both strains, may be partially attributable due to the obstruction in vision, because no significant inter-strain differences were observed, differences in rotarod performance are likely to be caused by another variable. CB6F1J mice exhibited higher body fat and lower lean mass percentages, potentially impacting their ability to maintain balance on the rotating rod. Although metabolic differences have been documented [9, 24–26], variations in maternal care could also contribute to observed disparities. Corder et al., 2023 utilized natural cross-fostering (i.e., trio breeding scheme of one sire and two dams per cage) rather than the typical littermate controls, to mitigate environmental and parental care discrepancies. While differences in metabolic activity were nullified, CB6F1J mice still showed inferior rotarod performance compared to C57BL/6J [6], signifying that body composition does not confound rotarod performance. Considering the role of brain structures in motor control may be a promising alternative. While Gandhay et al., 2023 identified age-related flattening of the hippocampus and pons in CB6F1J mice [4], no studies have evaluated these differences between CB6F1J and C57BL/6J strains. As age-related atrophy of the pons, which are involved in sensory processing and motor control, could be pivotal in explaining differences motor skills of between strains, further investigation into pons atrophy may elucidate the underlying reasons for these differences.

Given the substantial body of evidence linking anxiety to future cognitive decline [27–30], we conducted an analysis of anxiety-related behaviors using the open-field photobeam testing system to explore potential age-related strain differences. The open-field test (OFT) is a widely accepted method for assessing anxiety in mice, leveraging thigmotaxis, an instinctual tendency to avoid open spaces and intensive light due to predation vulnerability [31]. This behavior, deeply rooted in evolutionary survival mechanisms, is associated with challenges in emotional and spatial learning [32], with time spent in peripheral regions serving as a key anxiety parameter, quantified by the open field ratio (OFR). Our study revealed that 20- and 28-month-old C57BL/6J mice displayed significantly higher OFR compared to CB6F1J mice, consistent with a prior study from the same dataset showing lower overall movement in CB6F1J mice [33], indicating heightened anxiety in older C57BL/6J mice relative to their CB6F1J counterparts. While our findings align with previous observations of an inverse relationship between anxiety-like behavior and activity levels [34, 35], they diverge from existing literature suggesting lower baseline levels of anxiety in C57BL/6J mice without clear rationale [36, 37]. Additionally, CB6F1J mice exhibited age-related declines in both short-term and long-term memory (STM & LTM) while C57BL/6J mice did not, despite evidence suggesting independence between anxiety and memory impairment [33]. Given documented instances of memory impairment coinciding with anxiety symptoms [38–41], an unexplored variable in our study worth investigating further is the rate of adult neurogenesis (AN) between the two strains, which is shown to independently affect anxiety-like behaviors and cognition in rodents [42–44]. AN, the ongoing generation of neurons in specific brain regions like the ventral dentate gyrus (DG) of the hippocampus beyond early brain development stages [45], has been linked to anxiety-related behaviors and cognitive functions [31, 46–48]. Therefore, future studies should delve into these variables to unravel the intricate relationship between anxiety and cognition, particularly regarding potential strain disparities.

Both C57BL/6J and CB6F1J mice exhibited age-related cardiac dysfunction. Left ventricular hypertrophy (LVH) showed significant differences across all age groups, becoming more severe in 28-month-old mice, particularly in the CB6F1J strain, which exhibited a higher incidence and more severe cardiac dysfunction. This observation is supported by histopathological findings, revealing age-related cardiac lesions such as thickening and fibrosis of the tricuspid and pulmonary valves in CB6F1J, which were absent in C57BL/6J mice (unpublished findings). The aortic root to left atrial (AO/LA) ratio remained similar in both strains with aging, indicating age-related vessel thickening. While average diastolic and systolic blood pressures remained stable with age in both strains, CB6F1J mice showed a greater blood pressure elevation than C57BL/6J mice across all age groups. No significant associations were found between age or strain and other cardiac changes and functions (i.e., Ea/Aa ratio, IVCT T1, IVRT T3, MPI, FS%).

The cardiac aging process observed in mice closely mirrors human cardiac aging, characterized by cardiac hypertrophy, fibrosis, diastolic dysfunction, reduced functional reserve, and adaptive capacity to stress [49]. This parallel is crucial for understanding human cardiac aging, as the observed reduction in function contributes to the development of heart failure. However, selecting the appropriate animal model, such as rodents, and determining the relevant strain for specific studies and desired lesions is essential to avoid misinterpretation. Similar to humans, genetic background and family history play a significant role in cardiac function and heart health history, emphasizing the importance of careful consideration when using animal models for translational research. In this regard, CB6F1J mice would appear to be an excellent model to study age-related conditions involving function of the left ventricle.

Geropathology assessment of age-related lesions can provide useful information as to how drug treatment can effectively target organs and specific lesions within organs (3). Data from this study showed an age-related increase in lesion scores of the heart, liver, and kidney in both CB6F1J and C57BL/6J mice, as expected. The heart lesion scores reflected a similar increase in left ventricular dysfunction as already discussed. We did not conduct any specific liver function tests and found no associations between liver scores and metabolic data in either strain (p > 0.05). Since the liver is involved in a number of metabolic activities, additional consideration of the relationship between liver lesions and metabolic activities is warranted.

**Fig 11:**
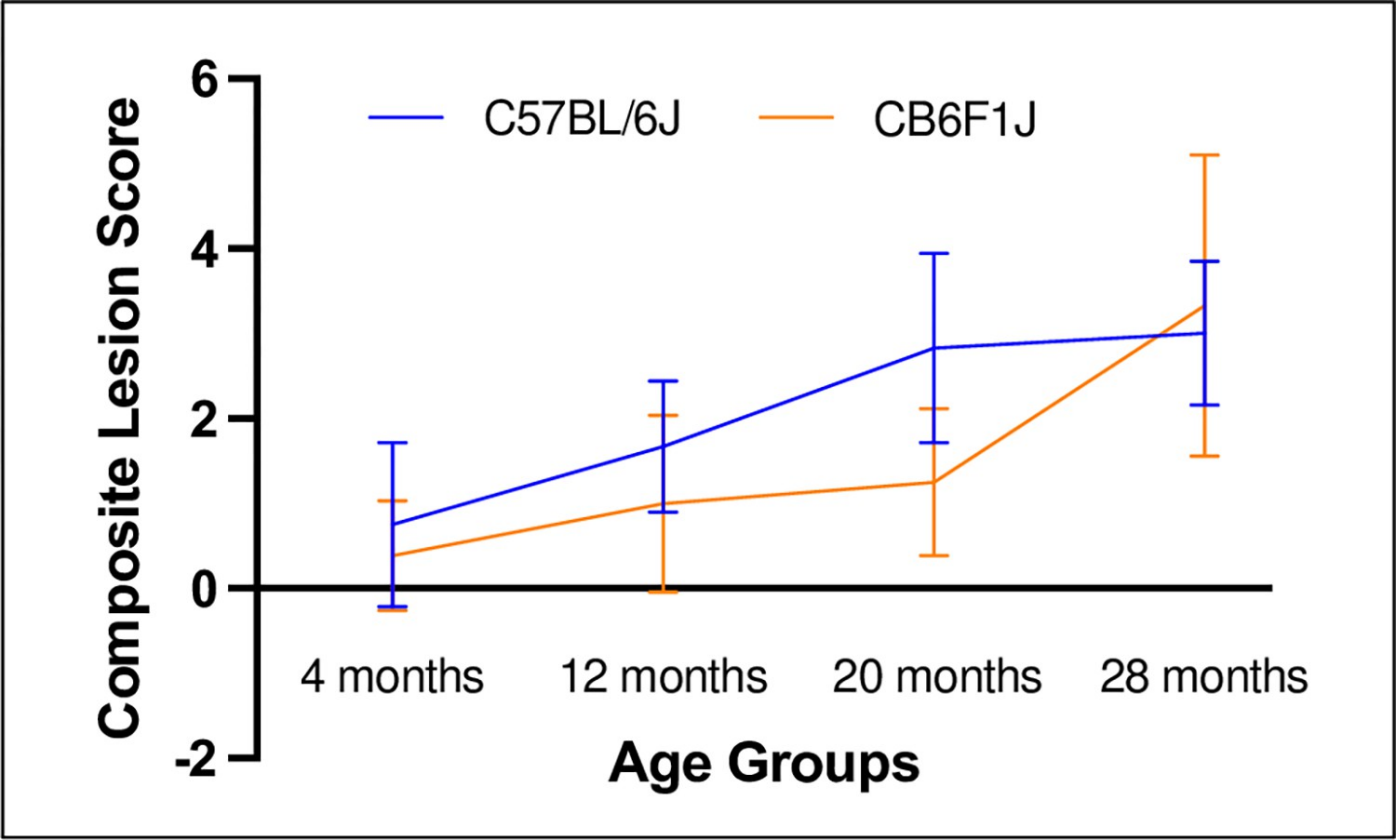
The kidney ages more rapidly in C57BL/6J mice compared to CB6F1J mice. CB6F1J mice at 24 months have kidneys with a pathobiological age of 16 months.

There was an age-related increase in lesion scores of the kidney that was more severe in C57BL/6J mice compared to CB6F1J. This is visually seen with increasing age in a graphic overview (Fig 11), which provides an example of how composite lesion scores (CLS) can distinguish lesion severity differences in the same organ from mice of the same age but of differing genetic backgrounds. Clearly, it can be seen that C57BL/6J mice attain a specific composite lesion score much earlier than CB6F1J mice. For example, at 24 months of age CB6F1J mice have a CLS similar to C57BL/6J mice at 16 months of age suggesting that the CLS system can be used to indicate the degree of pathological aging, a so-called “pathobiological clock”.

The age-related increase in lesion scores of the lungs in CB6F1J mice, but not C57BL/6J mice, is another example of how CLSs can distinguish differences in organ aging. We have previously reported that CB6F1J mice have a rather high incidence of primary lung tumors [50], which could be associated with the progression of lesion burden with increasing age in this strain, whereas C57BL/6J mice have very few primary lung tumors.

In summary, the genetic background of the two mouse strains influenced results of bioassays in an age-dependent manner. Therefore, it is imperative to recognize that different strains of mice may yield diverse data in preclinical aging studies and would need to be interpreted individually for translational applications. Both strains are readily available for aging studies from the Aged Rodent Colony subsidized by the National Institute on Aging. This provides the opportunity to obtain old mice to study naturally occurring age-related conditions such as chronic kidney disease, cardiomyopathy, lung cancer, hematopoietic tumors, cognitive decline associated with brain aging, anxiety-like behavior, and metabolic conditions. Which mouse strain to use will depend on the scientific focus and experimental objectives. Fig 11 summarizes differences and similarities observed in this study in male mice of both strains that may be helpful in initiating factors when considering either as a model for age-related studies.

## Supporting information

S1 File Geropathology lesion scores of major organs from C57BL/6 and CB6F1 male mice at 4 months, 12 months, 20 months and 28 months of age. (XLSX)
